# Green Tea Polyphenols and Sulfasalazine have Parallel Anti-Inflammatory Properties in Colitis Models

**DOI:** 10.3389/fimmu.2013.00132

**Published:** 2013-06-05

**Authors:** Helieh S. Oz, Theresa Chen, Willem J.S. de Villiers

**Affiliations:** ^1^Department of Internal Medicine, University of Kentucky Medical Center, Lexington, KY, USA; ^2^Department of Pharmacology and Toxicology, University of Louisville Medical School, Louisville, KY, USA; ^3^Division of Digestive Diseases and Nutrition, University of Kentucky Medical Center, Lexington, KY, USA

**Keywords:** IBD, enterocolitis, colitis, polyphenols, EGCG, sulfasalazine, IL-10^−/−^ mice

## Abstract

**Background:** There is no cure for autoimmune chronic inflammatory bowel disease (IBD). IBD patients commonly use complementary and alternative medications of which the safety, efficacy, and interaction with standard-of-care therapies are not fully known. Thus the consequences can become life-threatening. Sulfasalazine commonly used in IBD, potentially has severe adverse effects, including infertility, pulmonary fibrosis, lack of response, and ultimately patients may require intestinal resection. We hypothesized that green tea polyphenols (GrTP, EGCG) and sulfasalazine have similar anti-inflammatory properties.

**Methods:** BALB/c mice received Dextran sodium sulfate (DSS) to induce colitis (ulcerative colitis model). Exposure of IL-10 deficient mice (BALB/c-background) to normal microbiota provoked enterocolitis (mimics Crohn’s disease). Animals were treated with agents incorporated into daily diets. Control animals received sham treatment.

**Results:** DSS-treated animals developed severe bloody diarrhea and colitis (score 0–4, 3.2 ± 0.27). IL-10 deficient mice developed severe enterocolitis as manifested by diarrhea, rectal prolapse, and colonic lesions. Animals tolerated regimens (GrTP, EGCG, sulfasalazine) with no major side effects, and further developed less severe colitis. IL-10 deficient animals became moribund on high dose, while tolerated low and Mid doses with significant improved symptoms of enterocolitis. GrTP, EGCG, and sulfasalazine significantly ameliorated colonic damage and histological scores in treated animals in a similar manner (GrTP vs. DSS *p* < 0.05; EGCG, sulfasalazine vs. DSS *p* < 0.01). The inflammatory markers TNFα (3-fold), IL-6 (14-fold), and serum amyloid A (40-fold) increased in colitic animals and significantly decreased with treatment regiments. In contrast, circulatory leptin levels decreased in colitic animals (twofold). EGCG additionally reduced leptin levels (*p* < 0.01) while GrTP and sulfasalazine had no effect on leptin levels (*p* < 0.05). Hepatic and colonic antioxidants were significantly depleted in colitic animals and treatment regiments significantly restored antioxidants levels.

**Conclusion:** GrTP and EGCG improved antioxidants levels and attenuated severity of colitis analogous to sulfasalazine. Future studies will reveal whether polyphenols can become an alternative/additive therapy for IBD therapy in humans.

## Introduction

Crohn’s disease and ulcerative colitis are chronic idiopathic inflammatory bowel diseases (IBD) mediated by immune dysfunction. Despite advancement in humanized monoclonal antibodies and available targeted therapies, there is still no cure for IBD. Therefore, many IBD patients remain refractory to the existing therapies (Fiocchi, 2012). Furthermore, IBD predisposes patients to intestinal surgeries and colorectal malignancy. Inflamed colonic tissue in IBD patients (Grisham and Granger, 1988; Rezaie et al., 2007) and models (Oz et al., 2012a) are rich in neutrophils and activated macrophages and subsequent increased reactive oxygen (ROS) and nitrogen (NOS) species (Oz et al., 2012b). The excess generation of toxic radicals surpasses the intestinal antioxidant defensive ability, thus resulting in oxidative damage (Grisham and Granger, 1988; Oz et al., 2012a,b).

Sulfasalazine has been used as a mainstay of therapy in IBD for decades. Sulfasalazine is a prodrug composed of 5-aminosalicylic acid (5-ASA) and sulfapyridine linked by an azo bond that is poorly absorbed in the stomach and small intestine. The azo linkage is cleaved by the azoreductases released from terminal ileum and colonic anaerobic microbiota to form a pair of amines with the active moiety, 5-ASA (Scheline, 1973; Oz and Ebersole, 2008, review). Sulfasalazine acts as an antioxidant against generated ROS and NOS, with metal chelating effect which reduces oxidative burst. Sulfasalazine may protect against fibrosis by accelerating apoptosis in stellate cell (Oakley et al., 2005). In addition, sulfasalazine induces T lymphocyte apoptosis, inhibits inflammatory intermediates cyclooxygenase/lipoxygenase and nuclear factor kappa B (NF-kB) transcription pathway for pro-inflammatory cytokines, and activates peroxisome proliferator-activated receptor (Wahl et al., 1998; Cavallini et al., 2001; Liptay et al., 2002; Doering et al., 2004; Rousseaux et al., 2005).

However, sulfasalazine has a double edged sword effect by generating additional oxidative stress, which may result in hepatotoxicity (Uko et al., 2012) and ulcerogenic potential. Furthermore, sulfasalazine can provoke hypospermia, and male infertility (Linares et al., 2011), the underlying mechanisms are not fully understood (Katsanos et al., 2012). Sulfasalazine is shown to increase thiobarbituric acid-reactive substances (TBARS), and catalse activity while decrease superoxide dismutase and glutathione levels in hepatic, and kidney suggesting oxidative damage can be a mechanism for nephro-and hepatotoxicity and male infertility related to sulfasalazine treatment (Alonso et al., 2009; Linares et al., 2009).

Additionally, 5-ASA induces apoptosis of intestinal epithelia and inhibits regeneration of colitic mucosa (Reinacher-Schick et al., 2000; Brown et al., 2010). Some of these side effects (e.g., hepatotoxicity, and severe blood disorders) are due to the sulfapyridine portion of sulfasalazine. These patients require escalation of medical therapies and surgery. Therefore, safe and effective drugs are needed for this vulnerable population.

About 30–50% of IBD patients use some type of Complementary and Alternative Medicine (CAM) therapy (Opheim et al., 2012) in addition to their medications whether or not discussed with their primary care providers. However, the safety and efficacy of these compounds and interaction with other drugs in use have not been fully investigated. Therefore, the consequences can be potentially dangerous. The range of CAM therapies include: (i) hypnosis (Szigethy et al., 2011), (ii) acupuncture to decrease response to stress (Rawsthorne et al., 2012), (iii) megadoses of Vitamins and minerals, (iv) prebiotics (Oz and Ebersole, 2008; Oz et al., 2009) (v) probiotics (Mack, 2011), and (vi) Herbal Medicines (Geerling et al., 2000; Keefer et al., 2012). Amongst herbal therapy, tea and tea extracts have received a great deal of attention and are available over the counter (OTC). Tea (*Camellia sinensis*) is an evergreen shrub which has been used for about 4000 years and is the most consumed beverage after water (Mukhtar et al., 1992; Sharma et al., 2007). Tea contains several components including vitamins (B and C), minerals, and caffeine. Three types of tea are available depending on the processing technique. Black tea is produced by rolling and fermenting the leaves and consumed the most (78% consumption). Green tea is prepared from steamed and dried leaves (20% consumption). Oolong tea is an intermediate form when leaves are semi-fermented (2% consumption).

Green tea Polyphenols (GrTP) are antioxidants and we have previously shown them to have inhibitory effects on NF-kB *in vitro* in intestinal epithelial cells (Yang et al., 2001) and anti-inflammatory effects in IL-2 deficient mice and some aspects of dextran sodium sulfate (DSS) induced-colitis models (Varilek et al., 2001; Oz et al., 2005). GrTP are shown to have a variety of beneficial effects including anti colorectal cancer possibly through decreasing the serum levels of triglyceride (Shimizu et al., 2008) and promotion of apoptosis (Shirakami et al., 2008; Oz and Ebersole, 2010). In addition, GrTP blocks cyclooxygenase (Cox2) and BCL-2 activity to protect against acetaminophen hepatotoxicity (Oz and Chen, 2008; Oz et al., 2009), as well as LPS induced and carbon tetrahydrochloride hepatotoxicity (Chen et al., 2004). Polyphenols are broken down by the gut microbiota. Similarly, about 70–90% is excreted into feces and the rest recovered from urine (Griffiths and Smith, 1972). Polyphenols are the main component of green tea which have received extensive attention and contains four known catechins: (-)-epigallocatechin-3-gallate (EGCG), (-)-epigallocatechin (EGC), (-)-epicatechin-3-gallate (ECG), and (-)-epicatechin (EC). EGCG accounts for about 40% of the total polyphenols in tea.

We hypothesized that the alternative therapy with GrTP and EGCG protect against inflammatory responses in DSS induced ulcerative colitis and in the IL-10 deficient model of spontaneous enterocolitis (resembling Crohn’s disease) models in a dose dependent manner similar to the standard-of-care agent sulfasalazine.

## Materials and Methods

The animal studies were approved and performed in accordance with the guidelines for the care and use of laboratory animals accredited by the American Association of Accreditation of Laboratory Animal Care (AAALAC) at Veterans Administration (VA) and Laboratory Animal Research Resource Facility at the University of Kentucky Medical Center in Lexington, KY, USA. Animals were divided into groups of nine mice each (three/cage) and study was repeated at least once. All experiments conform to the relevant regulatory standards.

## Animal Models of IBD

### IL-10 deficient mouse model

Interleukin-10 deficient breeding pairs in BALB/c-background were originally obtained from Taconic/Dr. Rennick (Rennick and Fort, 2000) and bred in our transgenic facility. Animals were raised under microbial and pathogen-free conditions in ventilated microisolators with HEPA-filtrated air. Animals were handled in the biosafety cabinet with HEPA-filter and supplied with irradiated and autoclaved food, water, bedding, and cages.

In addition, 5-week-old male BALB/c (wildtype-background) mice were purchased from Harlan Laboratories (Indianapolis, IN, USA) and housed in micro-filter top cages and acclimatized for 1 week prior to the experiment. The IL-10 deficient mice were co-housed with wildtype mice in conventional condition, with free access to water and food (Harlan Teklad Laboratory Diet, Madison, WI, USA) and kept in a room with a 12 h light/dark cycle.

#### Colitis induction

##### Enterocolitis model

Enterocolitis was induced in IL-10 deficient mice by exposure to the normal gut microbiota. Therefore, IL-10 deficient male pups were weaned at 3 weeks of age, and at 4 weeks were transferred into the conventional facility in a room with unsterilized and filter top cages to reduce aerosolized contaminate. Cages were lightly seeded with contaminated (used) bedding from the same age BALB/c wildtype-background. This ensured rapid gut colonization with the microbiota, thus provoking intestinal inflammation and enterocolitis which mimic Crohn’s disease (Rennick and Fort, 2000; Oz et al., 2004).

##### Colitis model

Colitis was induced exclusively in BALB/c WT mice by oral ingestion of 3% DSS for seven consecutive days and the outcome was assessed by the clinical disease index, inflammatory mediators, and histological grading scores.

*Green tea polyphenols*. Green tea polyphenols containing >98% pure polyphenols analyzed with high-performance liquid chromatography (HPLC) were purchased from LKT Laboratories, Inc. (St. Paul, MN, USA). HPLC analysis of the GrTP extracts revealed the percentage composition of the polyphenols (four catechins) of interest as follow: Epicatechin-gallate (EC 35%), epigallocatechin (EGC 15%), epicatechin-gallate (ECG 4%), and epigallocatechin-gallate (EGCG 38%). The most prevalent individual polyphenolic constituent, EGCG (98% purity) attributed for GrTP therapeutic effects was purchased from Sigma Aldrich (St. Louis, MS, USA).

Sulfasalazine was purchased from Sigma Aldrich (St. Louis, MS, USA). Controls received vehicle sham treatment (sucrose). The compounds were incorporated into daily diet for the duration of the study (10 days for colitis). Animals consumed the diet with no significant difference compared the sham vehicle. DSS animals were treated with GrTP High (1%), or sulfasalazine (50 mg/kg) incorporated into diet. EGCG was calculated according the constituent of GrTP (∼40%) and given at different doses of High (0.5%), Mid (0.25%), and Low (0.12%) in daily diet. IL-10 deficient mice in conventional environment (as mentioned above) were treated with three different doses of GrTP at High (1%), Mid (0.5%), and Low (0.25%) for the duration of enterocolitis experiment. Sham control animals received sucrose alone. IL-10 deficient animals on High dose lost weight and became moribund, therefore were humanely eliminated. However, those IL-10 deficient mice on Mid and Low dose, tolerated the treatments for the 10 weeks duration of the study. At the end of experiments, animals were humanely euthanatized and blood and tissue samples were collected.

#### IL-10 deficient enterocolitis model (*n* = 9)

To establish the Enterocolitis Model

(A) Untreated normal mice kept in transgenic facility.
(B) Untreated mice exposed to normal gut microbiota in conventional faculty

GrTP and Enterocolitis Model

(A) Untreated mice exposed to normal gut microbiota in conventional faculty
(B) GrTP High dose (1%) treated mice exposed to normal gut microbiota in conventional faculty (were eliminated due to the morbidity)
(C) GrTP Mid dose (0.5%) treated mice exposed to normal gut microbiota in conventional faculty
(D) GrTP Low dose (0.25%) treated mice exposed to normal gut microbiota in conventional faculty

#### BALB/c mice and DDS induced-colitis model

GrTP/EGCG and BALB/c Mice (*n* = 3)

(A) Untreated normal mice
(B) GrTP High dose (1%) treated mice
(C) EGCG High dose (0.5%) treated mice
(D) EGCG Mid dose (0.25%) treated mice
(E) EGCG Low dose (0.12%) treated mice
(F) Sulfasalazine (50 mg/kg) treated mice

GrTP and DSS colitis model (n = 9)

(A) Untreated normal mice
(B) DSS-treated colitis mice
(C) DSS + GrTP High dose (1%) treated mice
(D) DSS + EGCG High dose (0.5%) treated mice
(E) DSS + EGCG Mid dose (0.25%) treated mice
(F) DSS + EGCG Low dose (0.12%) treated mice
(G) DSS + sulfasalazine (50 mg/kg) treated mice

*The clinical disease*. Animals were monitored for appearance, weight loss, consistency of stool, diarrhea, presence of blood in the stool, prolapse, survival, and anemia as expressed by the hematocrit, and colonic and splenic weight and length were measured.

*Blood and plasma isolation*. Immediately after euthanasia, blood was collected via the right ventricle of the heart into the lightly heparinized syringes and kept on ice. Plasma was separated by centrifugation at 5000 × *g* for 5 min at 4° C. Samples were stored at −80° C until further analysis.

*Colonic histopathology*. Colonic tissues were flushed with ice cold phosphate-buffered saline (PBS pH 7.2) and a portion from ascending and descending colonic tissues were fixed in 10% buffered formalin for histological examination. The remainder was snap-frozen in liquid nitrogen and stored at −80 °C. The formalin fixed sections were sliced at 5 μm then processed and stained with hematoxylin and eosin (H&E) and evaluated for the histopathology under light microscopy. Severity of colitis was assessed with a histological semi-quantitative grading score (Oz et al., 2005, 2007). The scores were based on histological features with a numeric value (0–4) assigned according to the tissue involvement and severity of lesions that corresponded to either of following criteria.

Grade 0 – No detectable lesions, no inflammatory cells, normal mucosal appearance.

Grade 1 – Few focal inflammatory infiltrate in the mucosa and lamina propria, epithelial hyperplasia (25% involved).

Grade 2 – Mild inflammation with a few multi-focal expansion of monocytes, neutrophils (PMN), epithelial hyperplasia into the mucosa (50% involved).

Grade 3 – Moderate inflammation with multi-focal expansion of mono, PMN, crypt abscess, epithelial hyperplasia into the mucosa (75% involved).

Grade 4 – Anal prolapse, severe diffused inflammation with crypt abscess, mono, PMN, transmural epithelium disruption, and ulceration few mucin (over 75% involved).

*Tissue preparation for antioxidant determination*. Tissue homogenates (10% w/v) were prepared in 5% metaphosphoric acid, using all-glass Tenbroeck homogenizers, and kept on ice (Chen et al., 2000; Oz et al., 2004). After standing for 20–40 min, the homogenates were centrifuged for 1 min (10,000 *g*) and the acid-soluble fractions were collected for measurement of sulfhydryl (SH) and disulfides (SS). GSH, GSSG, and other thiols, cysteine, and cystine were simultaneously quantified by HPLC with dual electrochemical detection (HPLC–DEC). Samples (20 μl) were injected on to a 250 × 4.6 mm, 5 μm particle, C_18_ column (Val-U-Pak HP, fully end-capped ODS; Chrom Tech Inc., Apple Valley, MN, USA). The injected samples were eluted isocratically with a mobile phase consisting of 0.1 M monochloroacetic acid, 2 mM heptane sulfonic acid (ion-pairing reagent), and 2% acetonitrile at pH 2.8 and delivered at a flow rate of 1 ml/min. The compounds were detected in the eluent with a Bioanalytical Systems model LC4B dual electrochemical detector, using two Au-Hg electrodes in series with potentials of −1.2 and 0.15 V for the upstream and downstream electrodes, respectively. Current (nA) was measured at the downstream electrode. Analytes were quantified from peak area measurements using authentic external standards.

*Inflammatory biomarkers immunoassays*. Cytokines were assayed in animals according the manufacture’s recommended protocol. The concentrations of IL-1β, IL-6, TNFα, and leptin were measured with Quantikine M ELISA kits obtained from R&D Company (Minneapolis, MN, USA). Serum amyloid A (SAA) analyzed with Kits from BioSource (Camarillo, CA, USA).

*Statistical analysis*. Data was analyzed using ordinary and repeated measures ANOVA. It was further analyzed by *post hoc* test (Tukey compared all pairs) for statistical difference using GraphPad Instat and Prism Software for Windows (San Diego, CA, USA). Statistical significance between groups considered to be significant was set at *p* < 0.05. Results are expressed as the mean ± SEM unless otherwise stated.

## Results

**Figure 1:**
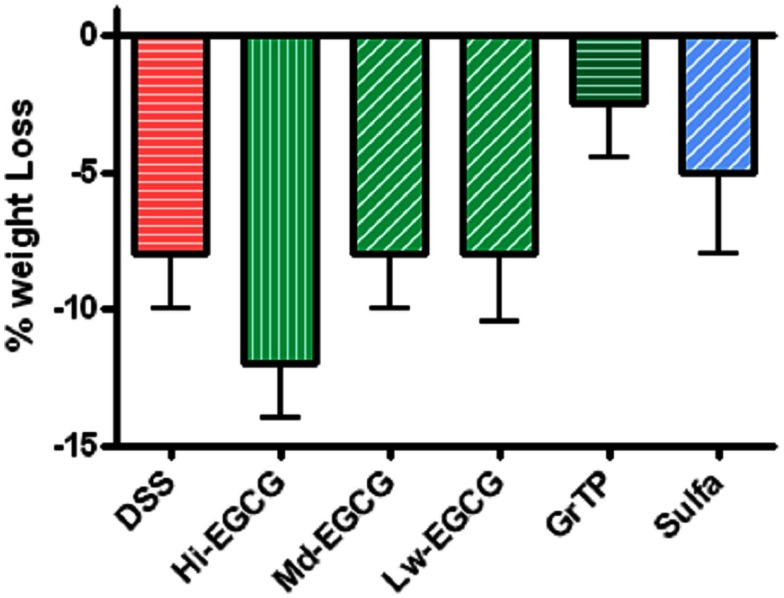
**Percent body weight loss in DSS-induced colitis compared to the normal control animals**. Colitic mice lost body weight and animals on High dose EGCG therapy showed the most weight loss. Mid and Low doses of EGCG had no effect on body weight. In contrast, GrTP and Sulfasalazine partially improved the body weight loss.

BALB/C wildtype animals tolerated GrTP, EGCG, and sulfasalazine in their daily diets with no severe side effects. Weight loss is a hallmark of colitis and colitic animals lost 8% of their body weight. GrTP (−2.5%) and Sulfasalazine (−5%) partially improved the weight loss in colitic animals, while, Mid and Low doses of EGCG had no effect on preserving animals’ weight (−8%). In contrast, High dose EGCG consumption further accelerated weight loss (−12%) (Figure 1). Colitic animals developed anemia due to bloody diarrhea, manifested with pale mucosa and a significant reduction in hematocrit (Control 41.5 ± 1.5 vs. colitic 25.2 ± 1.7 *p* < 0.05). EGCG and GrTP partially improved anemia and the hematocrit value. In contrast, sulfasalazine treatment further triggered anemia and the reduction in the hematocrit value (21.5 ± 2 *p* < 0.01) (Table 1). EGCG and GrTP administered to naïve control animals had no negative effect on the colonic weight or length. Colonic length became shortened (35%) and colonic weight increased in colitic animals due to the accumulation of inflammatory cells and EGCG partially improved the length (9%) with no effect on weight compared to colitic animals (Table 1). IL-10 deficient animals tolerated Low and Mid doses of GrTP and showed significantly improved enterocolitic symptoms while, lost weight and became moribund on high dose and were terminated.

**Table 1:** **Comparison of inflammatory markers and antioxidants between sham normal controls, DSS-induced colitic animals, and those treated with dose escalating EGCG or sulfasalazine**.

	Control	DSS	EGCG high	EGCG mid	EGCG low	Sulfa
Hematocrit	41.5 ± 1.5*^#^	25.2 ± 1.7*	31.9 ± 3.4	28.8 ± 3	30.9 ± 2	21.5 ± 2^#^
SAA μg/ml	8 ± 2^#^	327 ± 19^#^*	261 ± 44	258 ± 4*	298 ± 7	242 ± 53*
Colonic length	9.7 ± 0.6*	6.3 ± 0.2	6.9 ± 0.2	6.7 ± 0.1	6.9 ± 0.2	ND
Colonic weight	120 ± 5.7*	159 ± 9.0	166.8 ± 7.0	151 ± 4.5	154 ± 5.4	ND
Colonic GSH	2904 ± 462^#^*	168 ± 32^#^	1208 ± 405*	515 ± 215*	227 ± 63	147 ± 32^#^
Ileac GSH	ND	305 ± 118^^^	2443 ± 261^^^	2226 ± 163^^^	1338 ± 149*	600 ± 171^@^
Hepatic GSH	8188 ± 219	6283 ± 897*	7466 ± 235	5967 ± 407	6233 ± 565	6419 ± 280
Hepatic GSSG	433 ± 76	623 ± 99*	182 ± 49	400 ± 76	315 ± 106	386 ± 72
Hepatic ratio	19*^#^	10*	41^#^	15	20	17
Hepatic Cys	175 ± 9*	279 ± 14^#^*	148 ± 18^#^	246 ± 36	218 ± 9	241 ± 12*
Hepatic CSSC	51 ± 10	38 ± 7	39 ± 6	51 ± 14	46 ± 15	75 ± 11

*ND, not determined*.

*DSS administration reduced the Colonic length (<0.05) and increased the Colonic weight (<0.05) due to the inflammatory response compared the normal controls and treatments had no significant effects*.

*^@^Ileac GSH is partially increased by the sulfasalazine therapy but did not reach significance p = 0.05*.

*Oxidized (SS) and reduced (SH) sources of GSH are presented by nmol/g of tissue*.

*Hepatic Cys (cysteine) and CSSC (cystine) levels are presented by nmol/g of tissue*.

*DSS, dextran sodium sulfate-induced colitic animals*.

*Sulfa, sulfasalazine treated animals*.

*Hepatic ratio, represents ratio of liver reduced GSH/oxidized GSSG*.

*p < 0.05, ^#^p < 0.01, ^^^p < 0.001.

### Circulating inflammatory markers

**Figure 2:**
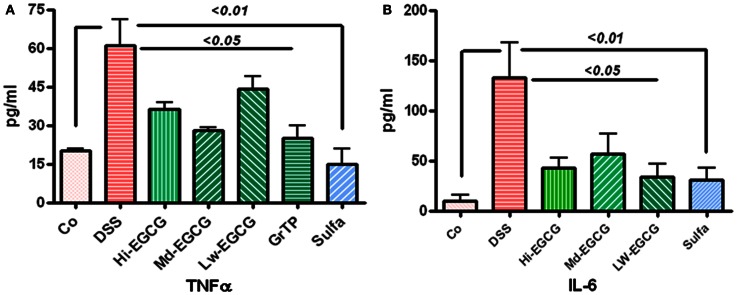
**(A)** DSS-induced colitic animals had increased secretion of inflammatory cytokine TNFα in blood circulation. EGCG therapy significantly prevented increased secretion (*p* < 0.05) and sulfasalazine normalized TNFα secretion. **(B)** Multifunctional cytokine, IL-6 was drastically increased in DSS-induced colitic animals. EGCG (*p* < 0.05) and sulfasalazine (*p* < 0.01) significantly reduced elevated level of this inflammatory marker in treated animals.

Colitis increased TNFα levels in blood circulation and sulfasalazine was most effective in normalizing TNFα release (vs. colitis *p* < 0.01). Sulfasalazine and to a lesser extent GrTP and EGCG (*p* < 0.05) decreased this pro-inflammatory cytokine (Figure 2A). Similarly, Blood levels of the multifunctional pro-inflammatory cytokine, IL-6 were significantly increased in colitic animals and EGCG and GrTP (*p* < 0.05) and sulfasalazine therapy (*p* < 0.01) significantly reduced secretion of IL-6 levels in treated animals (Figure 2B).

Serum amyloid A an inflammatory marker and an acute phase reactive protein was significantly increased in colitic animals (Control vs. colitic animals *p* < 0.01) and Mid dose EGCG and sulfasalazine partially but significantly (*p* < 0.05) ameliorated this circulating inflammatory marker (Table 1). GrTP therapy had a partial effect on the SAA which did not reach significance.

**Figure 3:**
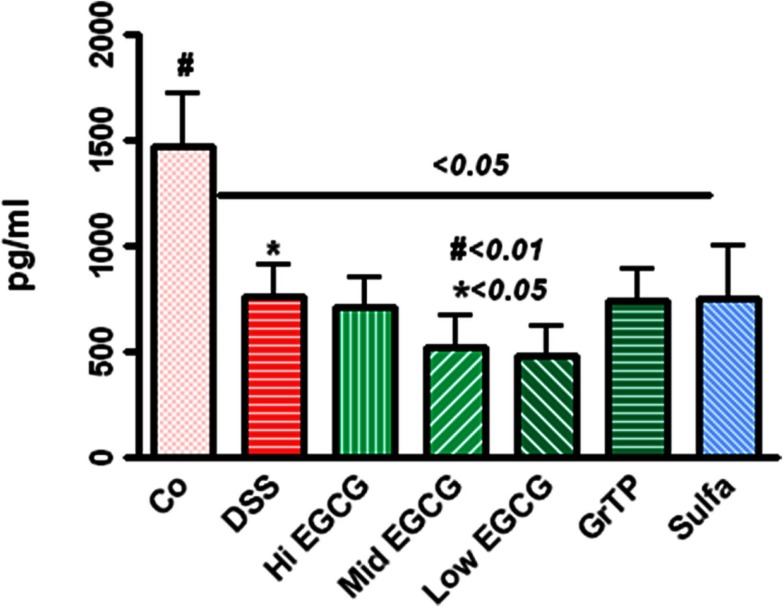
**Circulating leptin level significantly decreased in DSS-induced colitic animals (*p* < 0.05) and EGCG further reduced the leptin levels (DSS vs. EGCG < 0.05), while GrTP and sulfasalazine had no significant effect on leptin levels (vs. DSS > 0.05)**.

Leptin production, the marker of satiety, energy and expenditure, with central role in inflammatory response and immune defense was drastically decreased in colitic animals (*p* < 0.05) and EGCG consumption further reduced the leptin levels while GrTP and sulfasalazine had no additional affect on leptin regulation (*p* < 0.01) (Figure 3).

### Colonic lesions

**Figure 4:**
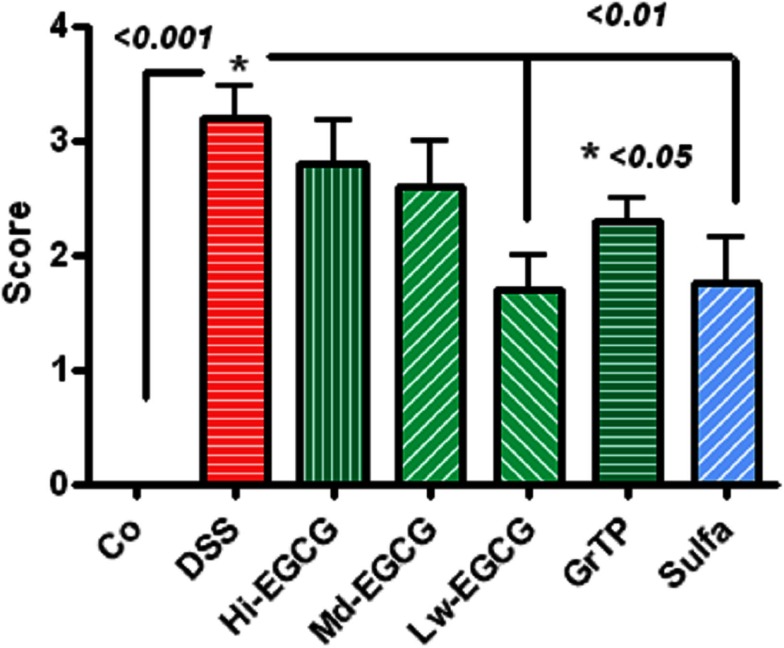
**Pathologic scores (zero-normal to four most severe) in colitic animals**. DSS-induced severe colonic pathology. Low dose EGCG and sulfasalazine similarly attenuated pathological lesions (*p* < 0.01). To a lesser extent, GrTP (*p* < 0.05) ameliorated the colonic lesions.

Dextran sodium sulfate-induced severe colitis manifested with infiltrations of immune and inflammatory cells including neutrophils and macrophages, loss of crypts, and ulcerations scored 3.2 ± 0.27 *p* < 0.001 (zero-normal control to four severe). Sulfasalazine and Low dose EGCG similarly (*p* < 0.01) and GrTP (*p* < 0.05) to a lesser extent attenuated the pathological lesions and preserved colonic microstructure (Figure 4).

### Antioxidant activity

Glutathione (GSH) is the most essential intracellular element to protect intestinal epithelial cells against ROS, and to preserve the gut integrity. Hepatic GSH (*p* < 0.01) which is the main source of gut antioxidant and colonic GSH (*p* < 0.05) was drastically depleted in the colitic animals. EGCG as well as GrTP significantly improved intestinal GSH. In contrast sulfasalazine had minor restorative effect on intestinal GSH (Table 1). The oxidized glutathione (GSSG) increased in colitic animals indicating accumulation of oxidative radicals in these organs and improved with therapies (Table 1). The ratio of hepatic reduced-to-oxidized glutathione, GSH/GSSG, was decreased to about 1/2 of the normal control levels indicating DSS-induced colitis as a global oxidative stress model. GrTP, Low dose EGCG, and sulfasalazine treatment similarly normalized hepatic glutathione concentration ratio. In contrast, High dose EGCG treatment resulted in drastic (fourfold) increases in the hepatic glutathione ratio, demonstrating exaggerated global antioxidant activity of the High dose EGCG (Table 1).

### GrTP and spontaneous enterocolitis

**Figure 5:**
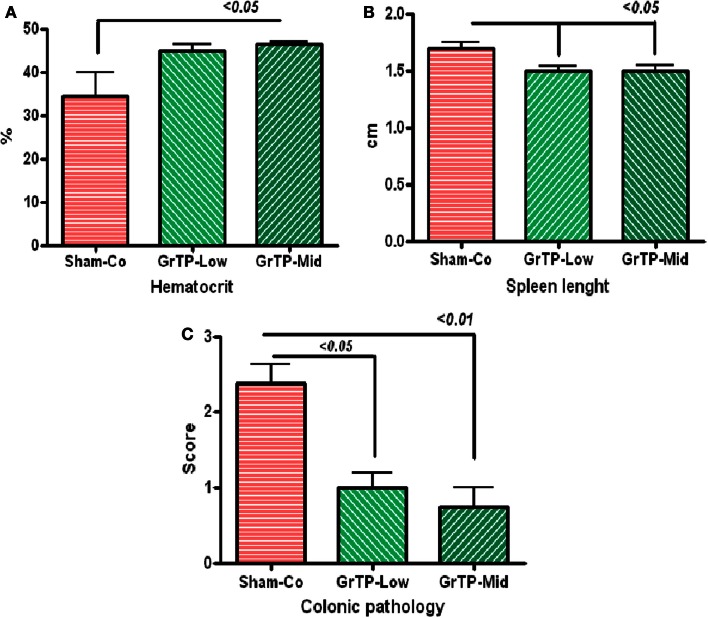
**IL-10 deficient mice when exposed to the normal colonic microbiota (Sham-Control) developed enterocolitis in conventional environment**. **(A)** IL-10 deficient mice became anemic with low hematocrit (Sham-Control) and GrTP significantly improved hematocrit in treated animals. **(B)** IL-10 deficient mice had enlarged splenic tissue due to infiltration of inflammatory cells and GrTP significantly reduced the inflammatory response. **(C)** IL-10 deficient mice developed spontaneous enterocolitis and rectal prolapsed. GrTP significantly ameliorated the pathological scores.

We further examined efficacy of GrTP against enterocolitis in IL-10 deficient mice exposed to normal gut microbiota. Animals became anemic (Figure 5A) and showed increased colonic weight (309 ± 39) and splenic length (1.7 ± 0.05) due to accumulation of inflammatory cells and enterocolitis (Figure 5B). IL-10 deficient animals tolerated Low and Mid doses of GrTP with significant improvement in their enterocolitis symptoms for the duration of experiment. While, animals on High dose lost weight and became moribund and were terminated. GrTP significantly improved anemia, decreased colonic weight (254 ± 17), and normalized splenic length (1.5 ± 0.04 *p* < 0.05). In addition, IL-10 deficient mice had significantly high SAA (100 times) and IL-1β (200 times) compared to those kept at transgenic conditions. GrTP had no significant effect on IL-1β with a partial effect on SAA in this model (data not shown).

Sham treated IL-10 deficient mice (Sham-Co) kept in the conventional environment developed severe enterocolitis manifested with moderately severe pathological lesions (score 2.4 ± 0.3) including infiltration of inflammatory and immune cells lamina propria, ulceration, and rectal prolapse when compared to those control littermates kept in transgenic environment (0.3 ± 02, data not clown). Colonic lesions were significantly improved in GrTP treated animals (Mid GrTP 0.75 ± 0.3 *p* < 0.01, and Low dose 1 ± 0.2 *p* < 0.05) when compare the sham controls demonstrating GrTP potential effect in treatment and/or maintenance in IBD models (Figure 5C).

## Discussion

Despite advances in humanized monoclonal antibodies and available targeted therapies, there is no cure yet for IBD. Biologic therapies, such as monoclonal antibody treatment are prohibitively expensive and have potential adverse effects including infections with fungi, JC virus, and tuberculosis. Many IBD patients also remain refractory to the existing therapies. Furthermore, IBD predisposes patients to intestinal surgeries and colorectal malignancy. Sulfasalazine is a standard care for treatment and maintenance in IBD also has severe adverse effects including hepatotoxicity (Uko et al., 2012), ulcerogenic and male infertility potential (Linares et al., 2011), as well as blood disorders in patients (Katsanos et al., 2012). Therefore, many of these patients seek CAM for symptom relief and improved quality of life.

Polyphenols are one of the most used herbal therapies available and have anti-inflammatory effect due to their antioxidant effects, alteration in the cell signaling, and particularly inhibition of the nuclear factor NF-κB pathway. Using IBD as a model of inflammation, we explored anti-inflammatory effects of the principal CAM, namely, GrTP and its most abundant cathechin EGCG, compared to the standard care, sulfasalazine treatment in murine colitis models. The susceptibility of mice to DSS-induced colitis and, polyphenols in specific EGCG-mediated anti-inflammatory action in this model have much in common with the similar phenomena observed with sulfasalazine.

While, colitic animals develop bloody diarrhea and anemia as IBD cardinal signs, GrTP and EGCG therapy were effective in improving hematocrit values. In contrast, sulfasalazine treatment further aggravated anemia in animals conceivably due to its adverse hemolytic effects as reported in IBD patients (Stein and Hanauer, 2000). In this study, EGCG was calculated according to the total amount of constituent present in the GrTP and administered at different doses of High (1), Mid (1/2), and Low (1/4) in daily diet. The Low dose EGCG appeared to be safe and to have the most effect on reducing colonic pathological lesions, normalizing global antioxidants ratio, partially improving colonic length and weight, without causing weight loss. However, Low dose was least beneficial in reducing SAA or in inhibiting reduction in leptin levels. While, GrTP normalized antioxidants, partially protected animals against weight loss and elevated TNFα but was less effective against colonic pathology as compared to Low dose EGCG.

In mice [3H](−)-EGCG is absorbed easily from the digestive tract and distributed widely into various organs, and excreted in the urine (6.4–6.6%) or feces (37.7–33.1%) within 24 h (Suganuma et al., 1998). In this study colonic GSH significantly decreased in colitic animals and improved in a dose dependent manner by EGCG treatment (DSS vs. High, Mid doses <0.05). However, sulfasalazine had no significant effect on the intestinal GSH. Of interest, the ratio of hepatic reduced-to-oxidized glutathione, GSH/GSSG, in colitic animals was decreased to about 1/2 of the amount in normal controls representing the DSS-induced colitis as a global oxidative stress model. Sulfasalazine, GrTP, and Low dose treatment normalized the hepatic glutathione ratio and the reversal of this important aspect of colitis in these models. Interestingly, High dose EGCG treatment caused fourfold increases in the hepatic glutathione ratio, demonstrating exaggerated global antioxidant imbalance presumably predisposing animals to excess weight loss with no improvement in colonic pathology.

In addition, EGCG and GrTP have been reported to have antimicrobial effects and to disrupt bacterial growth (Steinmann et al., 2013). In this study, GrTP and Low dose EGCG may have exerted their anticolitic effects through a combination of antimicrobial properties mucosal immunity and gut cleansing as well as antioxidants and anti-inflammatory action through inhibition of NF-kB activation and further IKK activity.

Leptin, an endocrine cytokine is a 16 kDa protein encoded by the *ob* gene which plays a central role in the maintenance of body weight and energy balance (Gaetke et al., 2002a). Leptin is secreted by the white adipocytes (Gaetke et al., 2002b, 2003) to regulate food intake and metabolism, while its action is controlled centrally by the hypothalamus (Zhang et al., 1994). Leptin regulates energy metabolism by increasing energy expenditure and decreasing food intake and body weight. The serum leptin concentration is linearly related to fat mass in *ad libitum* fed mice (Schwartz et al., 1997) and humans (Tuzun et al., 2004). Serum leptin rapidly declines with food restriction and is elevated with feeding (Schwartz et al., 1997; Gaetke et al., 2002a,b, 2003; Tuzun et al., 2004). Leptin deficiency affects both the innate and acquired immune systems (Mackey-Lawrence and Petri, 2012) as children and mice with congenital leptin or leptin receptor deficiency are reported to be susceptible to infections. The serum level of leptin is dysregulated in obesity (Schwartz et al., 1997) and during the active state of IBD (Tuzun et al., 2004). Leptin may present a potential mediator of inappropriate satiety and lipid dystrophy as well as deregulated immune response to altered gut microbiota in IBD patients. In this study colitic animals had significantly lower leptin levels. These findings are in accordance with leptin deficiency observed in other inflammatory diseases, including alcoholic hepatitis in mouse model (Tan et al., 2012). Additionally, Leptin has been shown to mediate resistance to enteric infection *Entamoeba* through its direct actions on intestinal epithelium which requires leptin receptor signaling through both the STAT3 and SHP2/ERK pathways (Guo et al., 2011). In the current study, GrTP and sulfasalazine had no effect on leptin levels. However, EGCG further reduced blood concentration of Leptin and High dose mediated additional weight loss possibly by blocking the appetite and decreasing food intake in colitic animals.

Our results have shown that 50 mg/kg sulfasalazine was sufficient to exert its protective effect against DSS-induced colitis. In contrast others reported that sulfasalazine at 10–20 mg/kg orally or IP did not prevent weight loss nor reduced diarrhea or gross pathology score in Trinitrobenzenesulfonic acid (TNBS)-induced colitis (Radi et al., 2011). This could be attributed to the insufficient drug dose used in the TNBS model against IBD.

Overall, these compounds had limited protective effects on DSS-induced colitis since colitis in general involves several key players including gut mucosal innate immune response, macrophage activation, ROS generation, and inflammatory response with subsequent loss of epithelia integrity, and increased luminal Gram-negative microbiota. Previously we and others have shown that GrTP inhibited signaling pathways involved in inflammation, including NF-kB in intestinal cells (Yang et al., 2001) and in IBD models (Varilek et al., 2001; Oz et al., 2005) and activated protein-1, AP-1(Abboud et al., 2008), which are key elements in production of pro-inflammatory mediators. Recently a mixture of EGCG and piperine was reported to protect against lipid peroxidation, neutrophils accumulation in DSS-induced colitic mice, while superoxide dismutase and glutathione peroxidase showed an increased activity in treated animals (Bruckner et al., 2012). To our knowledge this is the first report indicating: (a) GrTP to preserve gut microstructure and to protect against enterocolitis in IL-10 deficient model; (b) High dose EGCG consumption to provoke exaggerated global antioxidants and excess weight loss, rendering ineffective against colitis; (c) GrTP and Low dose EGCG to protect against colitis similar to the standard-of-care sulfasalazine, and to improve anemia.

## Conclusion

Green tea polyphenols and Low EGCG improved antioxidants pools, decreased inflammatory cytokines and attenuated the severity of colitis in a manner similar to that of sulfasalazine. Thus polyphenols may become an alternative/additive therapy for IBD therapy and future human clinical trials of the polyphenols in IBD patients are warranted.

## Conflict of Interest Statement

The authors declare that the research was conducted in the absence of any commercial or financial relationships that could be construed as a potential conflict of interest.

## Abbreviations

Cox-2, cyclooxygenase 2; DSS, dextran sodium sulfate; EGCG, (-)-epigallocatechin-gallate; GI, gastrointestinal tract; GrTP, green tea polyphenols; GSH, glutathione; IBD, inflammatory bowel disease; IEC, intestinal epithelial cells; IL-10, interleukin 10; LPL, lamina propria; LPS, lipopolysacchride; MΦ, macrophage; NF-κB, transcription nuclear factor kappa B; PBL, peripheral blood lymphocytes; ROS, reactive oxygen species; SAA, serum amyloid A; TNFα, tumor necrotic factor; WT, wildtype.
